# Faced with inequality: chicken do not have a general dosage compensation of sex-linked genes

**DOI:** 10.1186/1741-7007-5-40

**Published:** 2007-09-20

**Authors:** Hans Ellegren, Lina Hultin-Rosenberg, Björn Brunström, Lennart Dencker, Kim Kultima, Birger Scholz

**Affiliations:** 1 Department of Evolutionary Biology, Uppsala University, Norbyvägen 18D, SE-752 36 Uppsala, Sweden; 2 Department of Environmental Toxicology, Uppsala University, Norbyvägen 18A, SE-752 36 Uppsala, Sweden; 3 Department of Pharmaceutical Biosciences, Uppsala University, Box 594, SE-751 24 Uppsala, Sweden

## Abstract

**Background:**

The contrasting dose of sex chromosomes in males and females potentially introduces a large-scale imbalance in levels of gene expression between sexes, and between sex chromosomes and autosomes. In many organisms, dosage compensation has thus evolved to equalize sex-linked gene expression in males and females. In mammals this is achieved by X chromosome inactivation and in flies and worms by up- or down-regulation of X-linked expression, respectively. While otherwise widespread in systems with heteromorphic sex chromosomes, the case of dosage compensation in birds (males ZZ, females ZW) remains an unsolved enigma.

**Results:**

Here, we use a microarray approach to show that male chicken embryos generally express higher levels of Z-linked genes than female birds, both in soma and in gonads. The distribution of male-to-female fold-change values for Z chromosome genes is wide and has a mean of 1.4–1.6, which is consistent with absence of dosage compensation and sex-specific feedback regulation of gene expression at individual loci. Intriguingly, without global dosage compensation, the female chicken has significantly lower expression levels of Z-linked compared to autosomal genes, which is not the case in male birds.

**Conclusion:**

The pronounced sex difference in gene expression is likely to contribute to sexual dimorphism among birds, and potentially has implication to avian sex determination. Importantly, this report, together with a recent study of sex-biased expression in somatic tissue of chicken, demonstrates the first example of an organism with a lack of global dosage compensation, providing an unexpected case of a viable system with large-scale imbalance in gene expression between sexes.

## Background

The existence of males and females in sexually reproducing organisms and the associated difference in phenotypic optima between sexes imposes an intergenomic conflict in both the evolution of gene sequences and of gene expression. With the exception of the minority of the genome being confined to one sex, as for Y-chromosome sequences, there is thus a trade-off in the evolutionary genetic interests of the two sexes. One way organisms might respond to such sexual antagonism is to evolve sex-biased gene expression, in which the fixation of a sexually antagonistic allele (beneficial in one sex whilst being costly to the other) is followed by the evolution of modifiers to down-regulate gene expression in one sex [1]. It is increasingly recognized, using transcriptome profiling, that a significant proportion of the protein-coding genome has differential expression levels in males and females [2-4]. Many of these genes would be sex-biased in one or a few tissues only [4], so the total number of genes found to be sex-biased typically increases with number of tissues analysed; data from *Drosophila melanogaster *[3] and mice [4] indicate that as much as 50% of all protein-coding genes might be subject to sex-specific regulation of mRNA expression. Moreover, experimental work in *Drosophila melanogaster *confirms the frequent genomic occurrence of sexually antagonistic alleles and their response to selection [5,6].

In line with theoretical predictions for the probability of fixation of sexually antagonistic mutations [7], it has been observed that genes with sex-biased expression are non-randomly distributed in the genome. For example, male-biased genes expressed in somatic tissue of nematodes, flies and mammals are underrepresented on the X chromosome, and the same applies to genes expressed post meiosis in germ line [8-11]. In birds, male-biased genes are over-represented on the Z chromosome [12-14]. It has been shown experimentally in *Drosophila melanogaster *that the X is unusual when it comes to genes conferring sexual antagonism [15].

An obvious alternative explanation for the observation of sex-differential expression of sex-linked genes derives from the fact that gene dose differs between sexes. However, it is well known that organisms have evolved various mechanisms for equilibrating the expression of X-linked genes in males and females (dosage compensation), including X chromosome inactivation in mammals, up-regulation of gene expression on the single X chromosome of *Drosophila *males and down-regulation of gene expression of both X chromosomes of *Caenorhabditis elegans *hermaphrodites [16,17]. With the exception of individual genes that escape dosage compensation [18], sex-linked gene dose should therefore not be expected to lead to overall differences in expression levels between males and females. Intriguingly, in birds the status of dosage compensation is unclear [19,20]. Early work of sex-linked plumage traits in chicken and other bird species provided no evidence for a compensating mechanism [21], and this was followed by the landmark observation of a double dose of the Z-linked liver enzyme aconitase expressed in males compared to females [19]. Moreover, the absence of sex chromatin and the synchronous replication of the two Z chromosomes in males indicate that there is no Z chromosome inactivation [22,24,25]. More recent studies using real-time PCR experiments have added a further dimension to the question because the pattern that emerges is a heterogeneous one, with several examples of genes expressed at similar levels in the two sexes [23,25,26]. Importantly, a recent microarray-based study of global gene expression in somatic tissue has indicated that dosage compensation of sex-linked genes in chicken is less effective than is the case in mammals [27]. To study this in some further detail we have taken a genome-wide microarray approach to analyse sex-biased gene expression in both somatic tissue and gonads of chicken. Our data suggest that, overall, dosage compensation does not occur in chicken, meaning that the majority of sex-linked genes is expressed at lower levels in females than in males and that, in females but not in males, the expression levels of sex-linked genes are generally lower than of autosomal genes.

## Results and discussion

### Characterization of sex-biased gene expression in chicken

We used an oligonucleotide microarray (Affymetrix; 32773 transcripts, corresponding to over 28000 genes and thought to represent the majority of all protein-coding genes in the chicken genome) to measure expression levels in soma (brain and heart) and gonads (testes and ovary) of 18-day-old embryos. In total, there were 4665 out of 19743 (23.6%) surveyed genes with detectable expression that had a fold-change greater than 1.5 (corrected p < 0.05) between the two sexes. The proportion of genes showing sex-biased gene expression was highest in gonads 25.7% (4494/17438) followed by heart 1.8% (277/15398) and brain 1.7% (286/16846). Overall, there were about as many genes with higher expression level in males (2268) as there were in females (2408) (Additional files 1 and 2). Relatively few genes were differentially expressed in more than one tissue (Additional file 3).

### The genomic distribution of sex-biased genes

Sex-biased genes are non-randomly distributed in the chicken genome. The most pronounced deviation from random expectations is the disproportionate enrichment of male-biased genes on the Z chromosome (Table 1). The Z chromosome constitutes about 5% of the chicken genome [28] and the proportion of genes with detectable expression on the arrays being Z-linked was also 5.0%. However, 23.2% of all genes with >1.5 times (corrected p < 0.05) higher expression in males than in females were Z-linked (Fisher's exact test, p < 0.001). More than 80% of male-biased genes in brain (90.5%) and heart (87.7%) were Z-linked, whereas sex-linkage was seen for 21.4% of male-biased genes in gonads. In sharp contrast to the significant excess of male-biased genes, the Z chromosome showed a deficit for female-biased genes; overall, 2.3% of all genes with higher expression in females than in males were Z-linked (p < 0.001, Fisher's exact test; Table 1).

**Table 1:** Genomic distribution of genes showing sex-biased expression in chicken at >1.5 fold-change and a corrected p < 0.05 (number of genes in parantheses)

	Z obs	Z exp	A obs	A exp	*p *value*
Female-biased					
Brain	0.067 (1)	0.048	0.933 (14)	0.952	NS
Gonads	0.021 (28)	0.051	0.979 (1278)	0.949	<0.0001
Heart	0.111 (3)	0.048	0.889 (24)	0.953	NS
All tissues	0.023 (31)	0.050	0.977 (1341)	0.950	<0.0001
Male-biased					
Brain	0.905 (143)	0.0480	0.095 (15)	0.952	<0.0001
Gonads	0.214 (263)	0.051	0.786 (986)	0.949	<0.0001
Heart	0.877(121)	0.048	0.123 (17)	0.953	<0.0001
All tissues	0.232 (305)	0.050	0.768 (1009)	0.950	<0.0001

*Fisher's exact test. NS, not significant.

For a fold-change cut off of 1.5 (corrected p < 0.05), 479 out of 623 Z-linked genes on the array (75.2%) showed male-biased expression in at least one tissue. At a fold-change of 2, 47.3% were male-biased. While these results would be consistent with theoretical expectations for the genomic distribution of (at least partially) dominant mutations, the enrichment of male-biased genes on the Z chromosome is so pronounced that general dosage compensation must be questioned. In the case of female heterogamety, dominant gain-of-function male-advantageous mutations are expected to be favoured on Z because they are more often exposed to positive than negative selection, as Z is in males two-thirds of the time [7]. However, it seems unrealistic that the great majority of all genes on the avian Z chromosome would evolve under sexual antagonism. Moreover, while an active and probably selectively favourable transfer of genes between autosomes and the X chromosome via retroposition has been documented in mammals [29] and *Drosophila *[30], the inability of the reverse transcriptase encoded by the avian *CR1 *LINE element to copy polyadenylated mRNA has generally prevented retrogene movements in the avian genome [28].

### Absence of dosage compensation in gonads and soma

**Figure 1:**
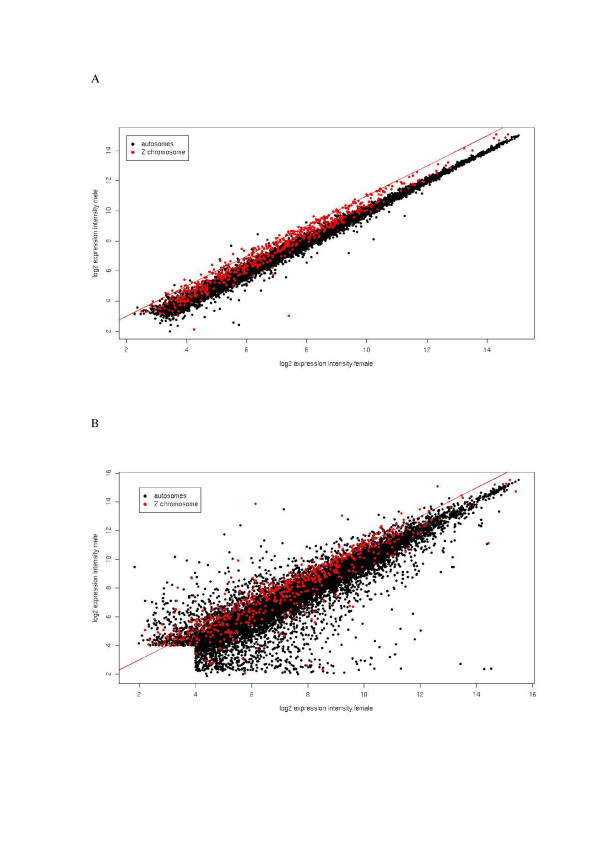
**Expression levels for individual Z-linked genes are higher in males than in females**. Scatter plots of the relationship between log_2 _hybridization intensities of individual genes in (a) soma and (b) gonads of male and female chicken embryos. The red line corresponds to twofold higher hybridization intensity in males than in females.

**Figure 2:**
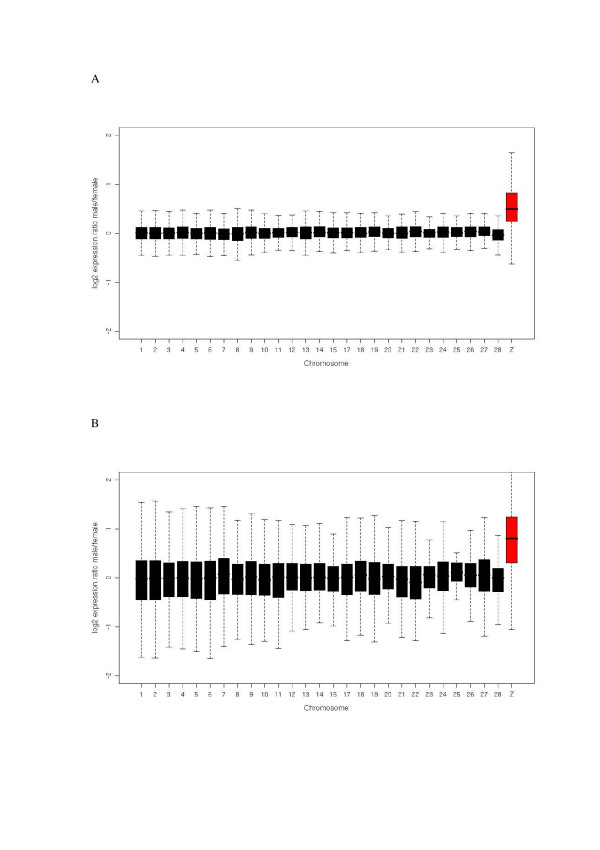
**Higher fold-change expression sex difference for the Z chromsome than for individual autosomes**. Box plots showing median of log2 male-to-female fold-change values per chromosome in (a) soma and (b) gonads. Boxes represent the mid 50% of the data (first and third quartiles) and whiskers extend to the minimum and maximum values that are not outliers (defined as >1.5 units away from first and third quartiles. Data from the Z chromosome is shown in red.

Male expression is higher than female expression for Z-linked genes over the whole range of observed expression levels, both for genes expressed in somatic tissue and in gonads (Figure 1). The mean fold-change of Z-linked genes is 1.42 for somatic tissue and 1.63 for gonads, which is quite different from individual autosomes that are all within a mean fold-change of 0.97–1.04 for soma and 0.88–1.09 for gonads (Figure 2). In the absence of large-scale dosage compensation, expression of Z-linked genes might be expected to be twice as high in males as in females. However, this assumes that gene dose is the only determinant of expression level, which is unlikely [31,32]. As pointed out by Gupta et al [33] and Zhang and Oliver [34], feedback regulation of biological networks will in many cases buffer differences in gene dose in that steady-state transcript levels and fold-change in expression become lower than gene dose difference. This is supported by observations that a global 1.5-fold gene dose difference due to trisomy or large chromosomal duplication in *Drosophila melanogaster *[33] as well as in mouse [35] result in a mean fold-change of only 1.1–1.2 in microarray experiments.

**Figure 3:**
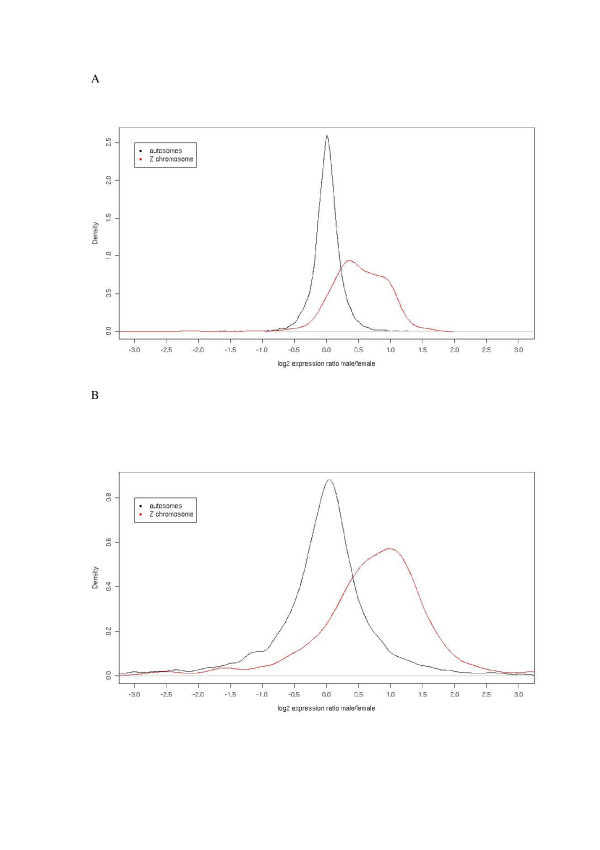
**The distribution of fold-change values differ between Z-linked and autosomal genes**. Fold-change values for autosomal (black) and Z-linked (red) genes in (a) soma and (b) gonads. Note different scales on y axes in (a) and (b).

The distribution of fold-change for individual Z-linked genes is shifted towards higher values, is broader and less symmetrical compared to the distribution for autosomal genes (Figure 3). This would be consistent with feedback regulation, or buffering, of Z-linked genes, the magnitude and efficiency of which must vary among genes involved with different pathways and networks. Moreover, the shape of the distribution of fold-change values for Z-linked genes tends to show a (secondary) peak at a fold-change of 2. This could reflect the fact that the regulation of expression of some genes is without feedback control mechanisms, yielding a close correlation between gene dose and expression level.

In a previous study we showed that microarray hybridization tend to underestimate sex-related differences in expression of Z-linked genes in chicken, as calibrated against data from real-time PCR experiments [14]. This would indicate that our mean fold-change values of 1.42 (soma) and 1.63 (gonads) probably reflect true fold-change values closer to 2. Importantly, these previous experiments give independent technical support for higher male than female expression of Z-linked genes in chicken. For nine Z-linked genes where microarrays revealed fold-change estimates of 1.20–1.64 (mean = 1.41) times higher expression in males than females, real-time PCR showed 1.44–2.12 (mean = 1.82) times higher male expression [14]. Moreover, there is a relatively strong correlation between fold-change estimates from microarray hybridization and real-time PCR (r^2 ^= 0.54, p < 0.01, Additional file 4). Moreover, a similar conclusion was reached by Itoh et al [27] who performed real-time PCR experiments with 18 zebra finch (*Taeniopygia guttata*) or chicken genes for which data on sex-biased expression was available from microarrays. The mean fold-change in their study was 1.55 for microarray data and 1.99 for real-time PCR (r^2 ^= 0.80, p < 0.001). In light of this, our data is most easily conceived in the absence of large-scale dosage compensation in chicken.

### Unbiased genes on the Z chromosome

**Figure 4:**
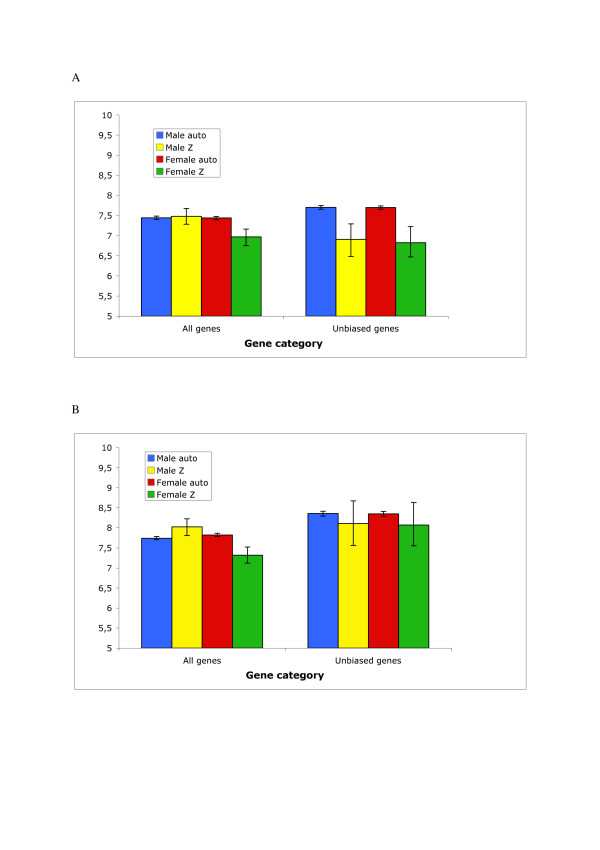
**Similar levels of male and female autosomal, and male but not female Z-linked, gene expression**. Histograms of mean log_2 _hybridization intensities for all genes, and unbiased genes (<1.2 fold-change), in (a) soma and (b) gonads. Male autosomal genes are shown in blue and female genes in red, whereas Z-linked genes in males are shown in yellow and in females in green. Error bars correspond to 95% confidence intervals.

It is of interest to specifically study the expression levels of sexually unbiased genes on the Z chromosome because this could potentially indicate how differences in sex-linked gene dose are being dealt with. Unbiased Z chromosome genes are expressed at lower levels than unbiased autosomal genes, for somatic tissue significantly so (≈ 1.8 times higher expression on autosomes than on the Z chromosome, p < 0.001) (Figure 4). This suggests that for unbiased Z-linked genes the major mechanism for sex-specific regulation is reduction of male expression. Furthermore, a number of gene ontology (GO) terms are significantly over-represented among unbiased Z chromosome genes (Additional file 5), while other terms are over-represented among those that are sex-biased (Additional file 6). For instance, genes with equal expression in males and females are in all tissues enriched for basic cellular functions such as regulation of metabolism and physiological processes.

In mammals, about 15% of X-linked genes escape dosage compensation through X chromosome inactivation [18] and it has been shown that genomic features such as the density of retrotransposon [36] and microsatellite repeats [37], sequence motifs [38-40], and CpG islands [18,41] correlate with the distribution of genes that are not repressed by epigenetic modification. Consistent with an absence of dosage compensation, we found no relationship between the degree and direction of sex-biased expression on the Z chromosome and neither GC content or the density of genes and *CR1 *retrotransposons (r^2^<0.01, not significant). However, fold-change values for individual genes are related to chromosome position with higher estimates on the q arm (mean gonads = 1.75, mean soma = 1.47) than on the p arm (mean gonads = 1.50, mean soma = 1.37, p < 0.005 in both cases). This suggests that the distribution of fold-change values over the Z chromosome is related to the evolutionary history of sex chromosomes. Similar to the case in mammals [42], avian sex chromosome evolution has been characterized by step-wise cessation of recombination between the Z and W chromosomes that have generated at least two evolutionary strata, roughly corresponding to the p and q arms [43]. Interestingly, the absence of dosage compensation would facilitate the fixation of male-beneficial mutations because the default situation is higher gene expression in males than in females. However, as the accumulation of sexually antagonistic mutations and the subsequent sex-specific regulation of expression levels are likely to be slow processes, the incidence of genes with pronounced sex-biased expression should increase with time since arrest of recombination between Z and W, which is observed herein.

### Unequal expression levels of sex-linked and autosomal genes in female birds

It has recently been recognized that gene expression from the single (active) X chromosome of mammals is up-regulated to harmonize levels of autosomal gene expression [33,44] and similar adjustment occur in worms and flies [33]. However, in female chicken, mean autosomal gene expression is 39% higher in somatic tissue (mean = 7.44. 95% CI = 7.39–7.48), and 43% higher in gonads (mean = 7.82, 95% CI = 7.78–7.86), than Z-linked gene expression (soma, mean = 6.97, 95% CI = 6.77–7.15, p < 0.001; gonads, mean = 7.32, 95% CI = 7.12–7.52, p < 0.001) (Figure 4, Additional file 7). Somatic expression levels of Z-linked genes in males are, on average, very similar to those of autosomal genes (mean log_2 _hybridization intensities of 7.48 (95% confidence interval (CI) = 7.28–7.68) and 7.44 (95% CI = 7.40–7.48), p = 0.37 by bootstrapping), while in gonads Z-linked expression is in fact somewhat higher (mean = 8.02, 95% CI = 7.82–8.22) than autosomal expression (mean = 7.74, 95% CI = 7.70–7.79, p = 0.006). In addition to the inequality in sex-linked gene expression between males and females, in chicken there is thus also an imbalance in sex-linked and autosomal gene expression in one of the sexes.

### Comparison to the study of Itoh et al [27]

Itoh et al [27] used a chicken oligonucleotide array and a small zebra finch cDNA array to demonstrate that expression levels of Z-linked genes in somatic tissue of these bird species are generally lower in females than in males. In the general sense, our study confirms the unexpected results of Itoh and co-workers [27], highlighting an unprecedented case of organisms in which the supposedly ubiquitous occurrence of dosage compensation of sex-linked genes is now shown to be weak or absent. However, there are also some differences between the two studies. The inclusion of gonads in the present study, but not by Itoh et al [27], is important because the proportion of genes showing sex-biased expression in gonads is much higher than in somatic tissue and because both the genomic distribution of sex-biased genes and the pattern of dosage compensation have been shown to differ between somatic tissue and germ line in other organisms [11,45]. Nevertheless, as for somatic tissue, we find that Z-linked gene expression in gonads is lower in females than in males, and that Z-linked expression in females is lower than autosomal expression. If anything, the difference between the sexes is more pronounced for gonads (mean fold-change of 1.63) than for somatic tissue (1.42).

Our study also adds other perspectives to the way the dose of sex-linked genes in chicken relates to expression levels. We show that, for genes on the Z chromosome that are not sex-biased, this is most likely achieved by down-regulation of male expression rather than up-regulation of female expression. We find that unbiased and sex-biased genes differ with respect to gene ontology, while there is no clear association between bias in gene expression and genomic parameters such as repeat content and base composition. However, there is a more distinct sex-bias in expression for genes on the evolutionarily older q arm than on the p arm.

Perhaps most importantly, while Itoh et al [27] concluded that dosage compensation is ineffective in birds, we question whether it occurs at all in the sense of a chromosome-wide mechanism for the general adjustment of expression levels of sex-linked genes in one or both of the sexes. As argued above, the observation of 1.4–1.6 times higher Z-linked expression in males than in females coupled with the facts that microarray hybridization tend to underestimate fold-change when calibrated against real-time PCR experiments and that negative feedback or autoregulation of expression is likely to occur at the level of individual genes, suggests to us that microarray data do not provide support for the existence of dosage compensation in birds.

## Conclusion

Chicken is the prime avian model. If we assume that the absence of large-scale dosage compensation in chicken is representative for other birds this obviously raises the question of how birds can cope with an imbalance in the expression levels of sex-linked genes in males and females. A similar question can be raised for the relative levels of gene expression of Z-linked and autosomal genes in females. As stated by Graves [20], "...the Z is a large chromosome, containing thousands of genes, and it would be remarkable if none of them were dosage-sensitive. An extra copy of most human autosomes is lethal in embryonic or fetal life". Possible explanations to this puzzling situation include that there would be less need for compensation, that there are constraints preventing large-scale compensation to evolve, or that compensation indeed does occur but at the post-transcriptional level.

One consequence of a lack of avian dosage compensation is that an excess of male-biased genes seen on the Z chromosome might not necessarily be related to the predictions from the fixation probability of sex-linked, sexually antagonistic mutations [12,13]. A critical test of these hypotheses would thus require other approaches than measuring the abundance of male-biased and female-biased genes on the Z chromosome.

The extensive sex difference in the expression of Z-linked genes would contribute to sexual dimorphism [26], which in many cases must be adaptive. It is noteworthy in this respect that sexual selection [46] and sexual dimorphism [47] are particularly pronounced in birds compared to many other organisms. Moreover, it is possible that large-scale differences in sex-linked gene expression between male and female birds are directly or indirectly related to avian sex determination, the mechanism of which is still a matter of debate. One model posits that Z chromosome dose determines sex in birds. If correct, absence of dosage compensation could either broadly affect the expression of critical components of the sex determining pathway or could be an indirect means for ensuring differential expression of a possible single sex-determining Z-linked gene, such as *DMRT1 *[48,49].

## Methods

### Sample collection

Fertilized eggs from White Leghorn fowl were purchased from OVA Production (Morgongåva, Sweden). The eggs were incubated at 37.5°C and 60% relative humidity, and were turned every 3 h. After 18 days of incubation (ed18), the embryos were euthanized by decapitation. A piece from the apical part of the heart was collected and the left gonad and the brain were excised. The cerebellum, the optic lobes and the cerebral hemispheres were removed from the brain and, consequently, the brain sample included the intact diencephalon and remaining parts from other regions. The samples were immediately frozen in liquid nitrogen and then stored at -70°C. The embryos were sexed by ocular inspection of the gonads and Müllerian ducts.

### RNA preparation and microarray hybridization

Heart, left gonads and diencephalon from each of four male and four female embryos were homogenized by syringe and needle followed by use of the Homogenizer system (Invitrogen, Carlsbad, CA, USA). RNA was extracted from heart and brain homogenates with the PureLink Micro-to-Midi Total RNA Purification System (Invitrogen), and RNA was then DNase treated with a DNA-free kit (Ambion, Austin, TX, USA). Due to limited amounts of available tissue, gonad RNA extraction was performed with an RNeasy Micro Kit (Qiagen, Hilden, Germany) with integrated DNase treatment. RNA concentration was measured with ND-1000 spectrophotometer (NanoDrop Technologies, Wilmington, DE) and RNA quality was evaluated using the Agilent 2100 Bioanalyzer system (Agilent Technologies Inc, Palo Alto, CA). A total of 2 μg of total RNA from each sample were used to prepare biotinylated fragmented cRNA according to the GeneChip^® ^Expression Analysis Technical Manual (Rev. 5, Affymetrix Inc., Santa Clara, CA, USA). Chicken Affymetrix   GeneChip expresssion arrays were hybridized for 16h in a 45°C incubator, rotated at 60 rpm. According to the GeneChip^® ^Expression Analysis Technical Manual (Rev. 5, Affymetrix Inc.), the arrays were then washed and stained using the Fluidics Station 450 and finally scanned using the GeneChip^® ^Scanner 3000 7G. In total, 24 hybridizations were made (4 individuals × 3 tissues × 2 sexes); however, three samples failed to meet Affymetrix quality control criteria and were removed from further analysis (heart and brain from one male, and gonads from one female).

### Microarray data analysis

All pre-processing and statistical analysis of microarray data was performed in R [50] version 2.4.1 using Bioconductor packages release 1.9 [51]. The CEL files were processed using GCRMA [52], a background adjustment method taking into account the GC content of probes when assessing non-specific binding, followed by quantile normalization and median-polish summarization of probe intensities into probe set intensities. A linear model was fitted to the log_2 _of the expression levels based on all probe sets and considering sex and tissue as a combined factor using the Limma package [53]. After pre-processing and linear model fitting the probe sets were filtered on expression; an expression threshold was set on both average expression level and absent/present calls from the R implementation of the Affymetrix MAS 5.0 algorithm. Only probe sets with average expression over a defined threshold and present in more than half of the samples within at least one tissue-sex combination were considered as significantly expressed. This resulted in 15398 probe sets for heart, 16846 for brain and 17438 for gonads, and these probe sets composed the reference for analysis of differently expressed genes between the sexes.

### Annotation of probe sets

Annotations for the probe sets were extracted from Ensembl [54] via biomRt in R. The Ensembl mapping of probe sets is based on alignments of individual probes to the chicken genome version 2.1 (WASHUC2 May 2006) and covers 21885 of the 37693 chicken-specific probe sets, which is close to the total number of protein-coding genes in the chicken genome identified by Ensembl. Several transcripts are represented by more than one probe set; the 21885 probe sets with annotation corresponds to 14414 unique transcripts. The genomic location for probe sets was taken from Ensembl. Gene ontology (GO) terms were available from the Gene Ontology Annotation Database [55] for 18239 of the 21885 chicken probe sets with Ensembl annotation. To get a broader overview of the GO terms assigned to genes, the terms were traced back to the ancestral term at different levels in each of the gene ontology classes biological process, molecular function and cellular component. These limited sets of terms where then used to test for over- and under-representation among biased genes.

### Statistical analysis

Fold-change was calculated as the average male expression over average female expression, and a Bayesian moderated t-statistic for differential expression between males and females was then generated for each tissue. To take multiple testing into account p values (corrected p values) were adjusted using the Benjamini and Hochberg false discovery rate (FDR) method [56]. Probe sets with an absolute fold-change value larger than various threshold levels were considered sex-biased. Statistical testing of differences in levels of hybridization intensities between sex and/or chromosome categories were performed by bootstrapping. The Fisher's exact test was used to test the distribution of gene ontology terms and the distribution of sex-biased genes across chromosomes. Spearman rank correlation coefficients were calculated for the continuous fold-change values and several genomic parameters including gene density, microsatellite and CR1 retrotransposons repeat density, and GC content, all taken from Ensembl. These parameters were estimated based on averaging over a 100 kb window surrounding each gene.

Microarray data presented in this study has been deposited to the Gene Expression Omnibus (GEO) at NCBI (Data set GSE8693).

## Competing interests

The author(s) declares that there a re no competing interests.

## Authors' contributions

HE conceived of and designed the study, analysed the data and wrote the paper, LHR processed and analysed the microarray data, BB dissected the birds, and LD, KK and BS participated in the design of the study.

## Supplementary Material

Additional file 1 Number of genes showing sex-biased expression pattern in brain, gonads and heart of chicken embryos at different cut-off levels. Click here for file

Additional file 2 Number of genes showing sex-biased expression in brain, gonads and heart of chicken embryos at different cut-off levels. Click here for file

Additional file 3 **Venn diagrams showing the number of genes with sex-biased expression in one or several tissues of chicken embryos**. (a) Genes with a fold-change of >1.5 and corrected p < 0.05; (b) genes with a fold-change of >2 and corrected p < 0.05. The total number of unbiased hybridizing genes is shown in the lower right corner. Click here for file

Additional file 4 **The relationship between observed male-to-female fold-change in expression level of Z-linked genes in microarray and real-time PCR experiments**. Data are from Scholz et al [14]. Click here for file

Additional file 5 Level 4–5 gene ontology (GO) terms for biological processes overrepresented among unbiased (fold-change <1.2) Z-linked genes. Click here for file

Additional file 6 Level 3–5 gene ontology (GO) terms for biological processes overrepresented among Z-linked genes showing male-biased expression (>1.5 fold-change, corrected *p *< 0.05). Click here for file

Additional file 7 **Box plots showing median of log_2 _absolute hybridization intensities in soma per chromosome in (a) females and (b) males**. Boxes represent the mid 50% of the data (first to third quartiles) and whiskers extend to the minimum and maximum values that are not outliers (defined as >1.5 times the box length away from first and third quartiles). Data from the Z chromosome is shown in red. Click here for file
