# Supplementary material for: Faced with inequality: chicken do not have a general dosage compensation of sex-linked genes
Source: BMC Biol. 2007 Sep 20;5:40. doi: 10.1186/1741-7007-5-40 (PMC2099419; doi:10.1186/1741-7007-5-40)
Supplement: Additional file 1 — Number of genes showing sex-biased expression pattern in brain, gonads and heart of chicken embryos at different cut-off levels. [file 1741-7007-5-40-S1.doc]

## Additional file 1 - Number of genes showing sex-biased expression pattern in brain, gonads and heart of chicken embryos at different cut-off levels

| Sex-biased pattern | Brain  (n = 16 846) | Gonads  (n = 17 438) | Heart  (n = 15 398) |
| --- | --- | --- | --- |
| Female-biased |  |  |  |
| > 1.5-fold | 603 (57) | 3 059 (2344) | 690 (75) |
| > 2.0-fold | 164 (40) | 1 808 (1660) | 232 (62) |
| > 2.5-fold | 90 (32) | 1 246 (1196) | 117 (50) |
| > 3.0-fold | 61 (29) | 978 (953) | 76 (41) |
| Male-biased |  |  |  |
| > 1.5-fold | 930 (229) | 2 947 (2150) | 845 (202) |
| > 2.0-fold | 282 (124) | 1 533 (1367) | 181 (77) |
| > 2.5-fold | 79 (25) | 927 (873) | 62 (29) |
| > 3.0-fold | 35 (11) | 644 (617) | 26 (11) |

The number of genes with corrected p < 0.05 is given in parentheses.
