# Supplementary material for: Faced with inequality: chicken do not have a general dosage compensation of sex-linked genes
Source: BMC Biol. 2007 Sep 20;5:40. doi: 10.1186/1741-7007-5-40 (PMC2099419; doi:10.1186/1741-7007-5-40)
Supplement: Additional file 2 — Number of genes showing sex-biased expression in brain, gonads and heart of chicken embryos at different cut-off levels. [file 1741-7007-5-40-S2.doc]

## Additional file 2 - Number of genes showing sex-biased expression in brain, gonads and heart of chicken embryos at different cut-off levels

| Fold-change* | Brain  (n = 16 846) | Gonads  (n = 17 438) | Heart  (n = 15 398) |
| --- | --- | --- | --- |
|  |  |  |  |
| > 1.5 | 1 533 (9.1%) | 6 006 (34.4%) | 1 535 (10.0%) |
| > 2.0 | 446 (2.6%) | 3 341 (19.2%) | 413 (2.7%) |
| > 2.5 | 169 (1.0%) | 2 173 (12.5%) | 179 (1.2%) |
| > 3.0 | 96 (0.6%) | 1 622 (9.3%) | 102 (0.7%) |
| > 1.5, p < 0.05 | 286 (1.7%) | 4 494 (25.7%) | 277 (1.8%) |
| > 2.0, p < 0.05 | 164 (1.0%) | 3 027 (17.4%) | 139 (0.9%) |
| > 2.5, p < 0.05 | 57 (0.3%) | 2 069 (11.9%) | 79 (0.5%) |
| > 3.0, p < 0.05 | 40 (0.2%) | 1 570 (9.0%) | 52 (0.3%) |

*Corrected p values (accounting for false discovery rate).
