# Supplementary figures and images for: Faced with inequality: chicken do not have a general dosage compensation of sex-linked genes
Source: BMC Biol. 2007 Sep 20;5:40. doi: 10.1186/1741-7007-5-40 (PMC2099419; doi:10.1186/1741-7007-5-40)

Additional File 3a


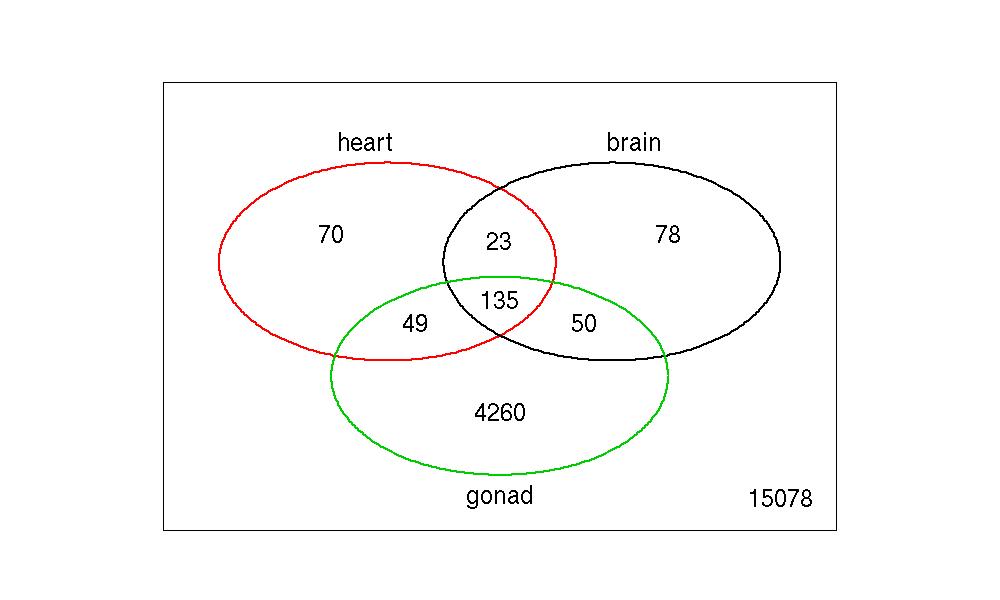


Additional File 3b


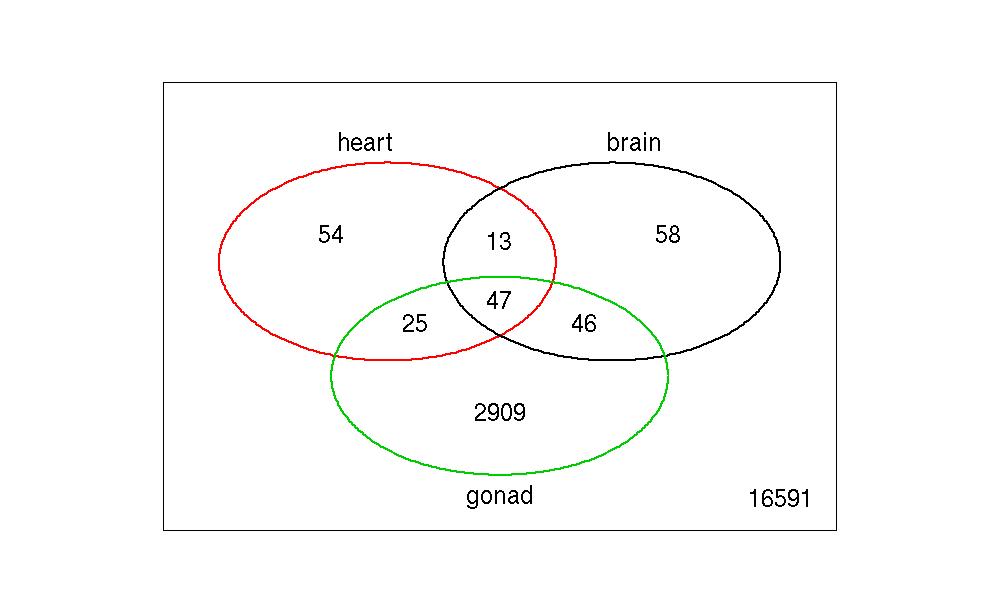

Supplement: Additional file 3 — Venn diagrams showing the number of genes with sex-biased expression in one or several tissues of chicken embryos. (a) Genes with a fold-change of >1.5 and corrected p < 0.05; (b) genes with a fold-change of >2 and corrected p < 0.05. The total number of unbiased hybridizing genes is shown in the lower right corner. [file 1741-7007-5-40-S3.doc]

Additional File 7a


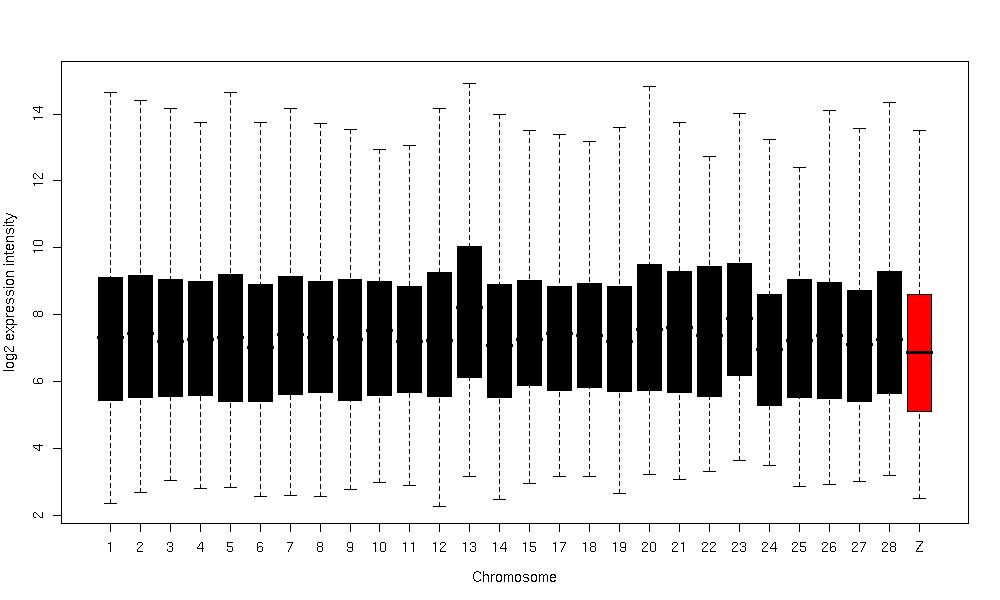


Additional File 7b


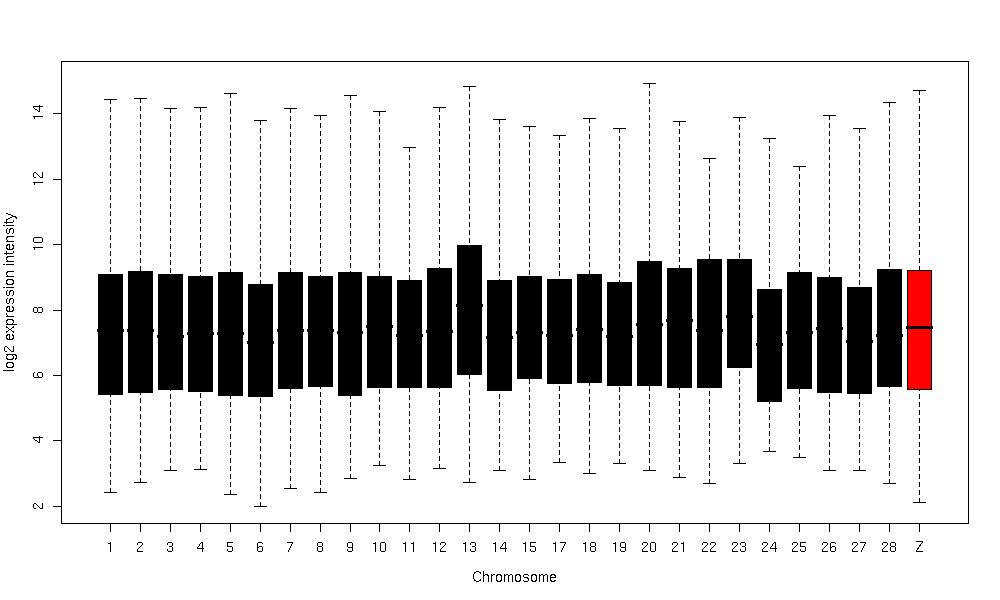

Supplement: Additional file 7 — Box plots showing median of log2 absolute hybridization intensities in soma per chromosome in (a) females and (b) males. Boxes represent the mid 50% of the data (first to third quartiles) and whiskers extend to the minimum and maximum values that are not outliers (defined as >1.5 times the box length away from first and third quartiles). Data from the Z chromosome is shown in red. [file 1741-7007-5-40-S7.doc]
