# Supplementary material for: Faced with inequality: chicken do not have a general dosage compensation of sex-linked genes
Source: BMC Biol. 2007 Sep 20;5:40. doi: 10.1186/1741-7007-5-40 (PMC2099419; doi:10.1186/1741-7007-5-40)
Supplement: Additional file 5 — Level 4–5 gene ontology (GO) terms for biological processes overrepresented among unbiased (fold-change <1.2) Z-linked genes. [file 1741-7007-5-40-S5.doc]

## Additional file 5 - Level 4–5 gene ontology (GO) terms for biological processes overrepresented among unbiased (fold-change < 1.2) Z-linked genes

| Tissue | Term name | GO level | p Value* |
| --- | --- | --- | --- |
| Gonads |  |  |  |
|  | Regulation of cellular physiological process | 4 | 0.004235 |
|  | Regulation of metabolism | 4 | 0.005499 |
|  | Organelle organization and biogenesis | 5 | 0.014653 |
|  | Negative regulation of cellular process | 4 | 0.019591 |
|  | Cellular metabolism | 4 | 0.027081 |
|  | Cell organization and biogenesis | 4 | 0.031598 |
|  | Regulation of cellular metabolism | 5 | 0.046449 |
| Brain |  |  |  |
|  | Regulation of metabolism | 4 | 0.021919 |
|  | Regulation of cellular physiological process | 4 | 0.025423 |
|  | Regulation of cellular metabolism | 5 | 0.035031 |
| Heart |  |  |  |
|  | Regulation of cellular physiological process | 4 | 1.43E-05 |
|  | Regulation of metabolism | 4 | 0.000181 |
|  | Regulation of cellular metabolism | 5 | 0.000686 |
|  | Nucleobase, nucleoside, nucleotide and nucleic acid metabolism | 5 | 0.04288 |

*Fisher’s exact test.
