# Supplementary material for: Faced with inequality: chicken do not have a general dosage compensation of sex-linked genes
Source: BMC Biol. 2007 Sep 20;5:40. doi: 10.1186/1741-7007-5-40 (PMC2099419; doi:10.1186/1741-7007-5-40)
Supplement: Additional file 6 — Level 3–5 gene ontology (GO) terms for biological processes overrepresented among Z-linked genes showing male-biased expression (>1.5 fold-change, corrected p < 0.05). [file 1741-7007-5-40-S6.doc]

## Additional file 6 - Level 3–5 gene ontology (GO) terms for biological processes overrepresented among Z-linked genes showing male-biased expression (>1.5 fold-change, corrected p < 0.05)

| Tissue | Term | Level | p Value |
| --- | --- | --- | --- |
| Heart |  |  |  |
|  | Response to endogenous stimulus | 3 | 0.014064 |
|  | Response to stress | 3 | 0.021695 |
|  | Cellular localization | 4 | 0.00497 |
|  | Response to DNA damage stimulus | 4 | 0.012292 |
|  | Intracellular transport | 5 | 0.004828 |
|  | Establishment of cellular localization | 5 | 0.004828 |
|  | DNA repair | 5 | 0.011967 |
| Brain |  |  |  |
|  | Metabolism | 3 | 0.012307 |
|  | Cell organization and biogenesis | 4 | 0.022342 |
|  | Macromolecule metabolism | 4 | 0.023866 |
|  | Cellular metabolism | 4 | 0.043105 |
|  | Macromolecule biosynthesis | 5 | 0.005805 |
|  | Cellular macromolecule metabolism | 5 | 0.008937 |
|  | Protein metabolism | 5 | 0.019372 |
|  | Intracellular transport | 5 | 0.033421 |
| Gonads |  |  |  |
|  | Establishment of cellular localization | 5 | 0.033421 |
