# Supplementary material for: Faced with inequality: chicken do not have a general dosage compensation of sex-linked genes
Source: BMC Biol. 2007 Sep 20;5:40. doi: 10.1186/1741-7007-5-40 (PMC2099419; doi:10.1186/1741-7007-5-40)
Supplement: Supplementary file 6 [file 1741-7007-5-40-S4.doc]

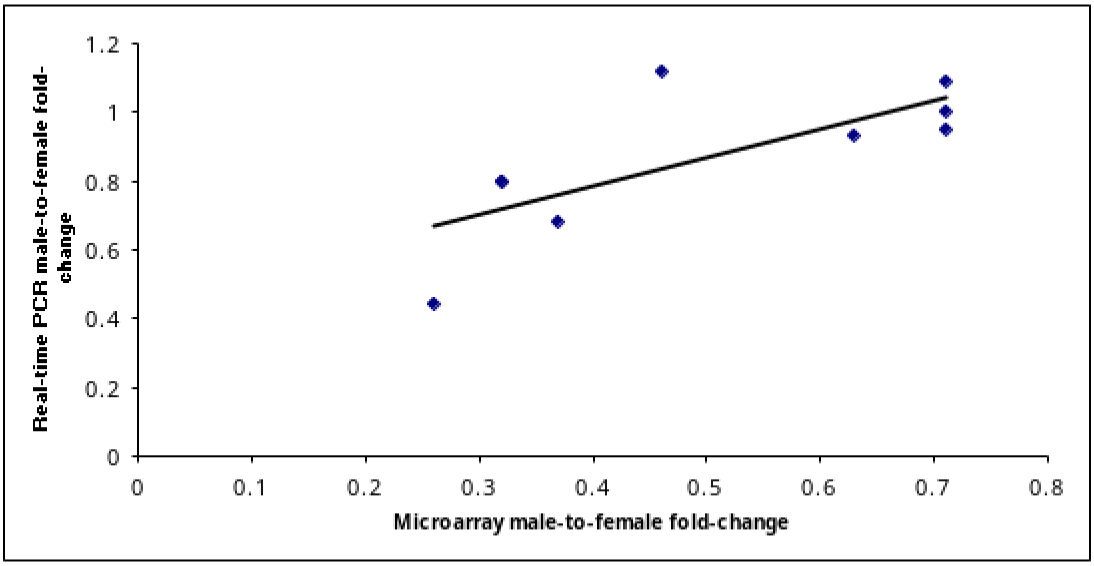

Real-time PCR male-to-female fold-change
Microarray male-to-female fold-change
1.2
1
0.8
0.6
0.4
0.2
0
0.1
0.2
0.3
0.4
0.5
0.6
0.7
0.8
